# Self-Stabilized Precipitation Polymerization and Its Application

**DOI:** 10.1155/2018/9370490

**Published:** 2018-09-10

**Authors:** Zhenjie Liu, Dong Chen, Jinfang Zhang, Haodong Liao, Yanzhao Chen, Yingfa Sun, Jianyuan Deng, Wantai Yang

**Affiliations:** ^1^College of Materials Science and Engineering, Beijing University of Chemical Technology, Beijing 100029, China; ^2^State Key Laboratory of Chemical Resource Engineering, Beijing University of Chemical Technology, Beijing 100029, China

## Abstract

An effective, value-added use of the large amounts of olefinic compounds produced in the processing of petroleum, aside from ethylene and propylene, has been a long outstanding challenge. Here, we developed a novel heterogeneous polymerization method, beyond emulsion/dispersion/suspension, termed self-stabilized precipitation (2SP) polymerization, which involves the nucleation and growth of nanoparticles (NPs) of a well-defined size without the use of any stabilizers and multifunctional monomers (crosslinker). This technique leads to two revolutionary advances: (1) the generation of functional copolymer particles from single olefinic monomer or complex olefinic mixtures (including C4/C5/C9 fractions) in large quantities, which open a new way to transform huge amount of unused olefinic compounds in C4/C5/C9 fractions into valuable copolymers, and (2) the resultant polymeric NPs possess a self-limiting size and narrow size distribution, therefore being one of the most simple, efficient, and green strategies to produce uniform, size-tunable, and functional polymeric nanoparticles. More importantly, the separation of the NPs from the reaction medium is simple and the supernatant liquid can be reused; hence this new synthetic strategy has great potential for industrial production.

## 1. Introduction

Radical-initiated heterogeneous polymerization, including emulsion, dispersion, and suspension polymerization, accounts for 25% of the production of synthetic polymers [1–3], due to the ease of heat removal and polymer products separation. For these heterogeneous polymerizations, emulsifiers or polymeric surfactants are utilized to stabilize the monomer droplets or latex particles, in which most of the polymerization reaction takes place. For example, for conventional emulsion polymerization [4], miniemulsion polymerization [5], and microemulsion polymerization [6], the formation and growth of polymer particles occurred mainly inside micelles formed by emulsifier. Suspension polymerization can prepare polymer particles with larger size, and the formation of particles depended on the violent agitation and protection of dispersant [2, 7, 8]. As to dispersion polymerization, although the reaction belongs to solution polymerization before the stage of the nucleation [9], the polymerization and polymer particle growth occur in the particles with the protection of amphiphilic surfactant or stabilizer formed in situ [10].

The above-mentioned approaches are well suited to particle formation, and there are various applications relying on their ability to control the uniformity, dimension, and shape of the particles produced [11, 12]. It is worth mentioning that the polymerization within the monomer droplets/micelles experiences a transition from liquid to solid, and, consequently, it is difficult to directly obtain uniform particles. Many modified approaches have been reported to overcome these drawbacks, including successive seeded emulsion polymerization and activated-swelling suspension polymerization [13, 14], one-step dispersion polymerization with a special graft copolymer as a stabilizer [15], two-stage dispersion polymerization [16], two-step emulsion polymerization plus macromonomer [17], and two-stage living radical dispersion polymerization [18]. However, emulsifiers or polymeric surfactants used in these heterogeneous polymerizations not only affect the purity and performance of the products but also cause serious water-pollution problems [1].

To overcome the disadvantage of surfactants, some precipitation polymerizations without dispersion agents were reported and narrow or even monodisperse polymer particles with complex structures could be successfully obtained [19]. However, most of them suffer from low monomer concentration (e.g., <5 wt%) and/or require special apparatus or processes (e.g., rotating reactor or distillation) to avoid the coagulation of the resultant polymer particles [20, 21]. The limitation on monomer loading greatly hindered the practical application of precipitation polymerization. In recent years, several new approaches have been developed to overcome the limitation on monomer concentration, including photoinitiated precipitation polymerization, solvothermal precipitation polymerization, and redox-initiated precipitation polymerization [22–24]. Additionally, it was reported that uniform polyurea (PU) microspheres were prepared with high productivity through precipitation polymerization using isophorone diisocyanate as the only monomer in mixed solvent of water-acetonitrile, though this process is limited to step growth polymerization of diisocyanate for the preparation of PU microspheres [25]. Although clean polymer particles free from surfactants can be successfully obtained at high monomer loading, there are still some inherent shortcomings for these newly developed methods. Due to high content of crosslinker in the reaction system, the as-prepared particles are usually crosslinked with high degree of crosslinking. Another thing that needs to be addressed is that the reaction time for photoinitiated and redox-initiated precipitation polymerization is too long (>48h), and the monomer conversion and particle yield are relatively lower (<40%).

Currently, slurry-phase and gas-phase polymerization in the presence of a heterogeneous catalyst (Ziegler–Natta, metallocenes, etc.) are the most commonly used processes for the production of polyolefins, including high-density polyethylene (HDPE), isotactic polypropylene (IPP), and their copolymers with higher olefins [26]. For this type of heterogeneous polymerization, the catalyst particles (10–50 *μ*m) and agglomerates of nanoscopic particles adsorb and polymerize gaseous monomers, producing insoluble polymer NPs that continuously grow to the micron scale [27]. The advantage of this heterogeneous polymerization is the ease in obtaining solid polymeric products. But there are two major limitations: (a) it is difficult to prepare polar or functional copolymers and (b) it is difficult to get well-defined nano- or micron-sized polymeric particles.

Here, we present a novel heterogeneous polymerization strategy, termed self-stabilized precipitation (2SP) polymerization, where the alternating copolymerization of olefinic compounds with maleic anhydride (MAH) or its derivatives shows a nucleation and growth of NPs with well-defined size. Most surprisingly, the resultant NPs are buoyant in the reaction medium without the help of surfactants. The polymerization is (a) a one-step precipitation polymerization producing functional copolymers of single olefinic compounds from the C4, C5, and C9 fractions; (b) a one-step precipitation polymerization for the preparation of functional copolymers using the C4, C5, or C9 fractions without separation; and (c) a route for the formation of NPs having a well-defined size, shape, and functionality. The solid polymeric NPs are easily separated by centrifugation or filtration, and the supernatant liquid can be repeatedly reused, making this strategy “green.” Due to the absence of any stabilizers and multifunctional monomers (crosslinker), high monomer concentration, and ease of product separation, 2SP can be scaled to the industrial level.

## 2. Results

### 2.1. Self-Stabilized Precipitation (2SP) Polymerization

We use the well-known radical alternating copolymerization of MAH and styrene (St) as a model reaction system to describe 2SP polymerization. When isoamyl acetate (IA) is used as the solvent and 2,2′-azobis (isobutyronitrile) (AIBN) as an initiator, the copolymerization of MAH and St proceeds smoothly, forming a stable colloidal dispersion of PMS particles. SEM, FT-IR, ^13^C NMR, and titration were utilized to characterize the resultant copolymer particles. It was found that the poly(maleic anhydride-*alt*-styrene) (PMS) copolymer particles were uniform in size with a chemical composition of (MAH/St=50/50) for all molar feed ratios of MAH/St, as shown in [Sec supplementary-material-1] and [Sec supplementary-material-1]. Since it is impossible to form PSt homopolymer at high molar feed ratios of MAH/St, the possibility of St homopolymer acting as a stabilizer can be excluded. Because there were not any stabilizers or in situ generated polymeric surfactants, we named this polymerization system as 2SP polymerization. Moreover, the monomer concentration could be as high as 40% (wt/v), offering great potential for industrial applications.

**Figure 1:**
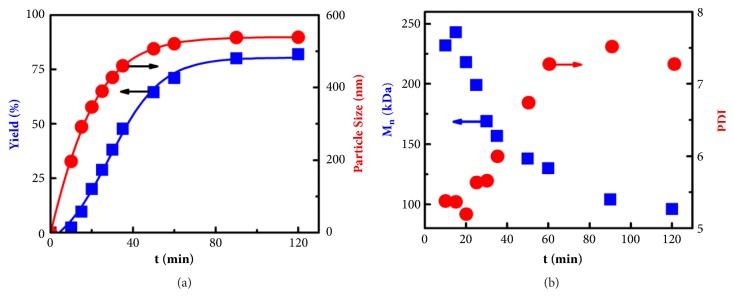
**Characterization of the obtained PMS particles.** The variation of** (a)** particle yield and diameter;** (b)** Mn and PDI of the PMS particles as a function of reaction time.

To get key mechanistic insight into the polymerization process, copolymerization of MAH/St at equal molar feed ratio was performed in IA with 10% (wt/v) monomer concentration and 0.06 wt% AIBN (relative to monomers). By heating to 70°C, the initially transparent solution turned turbid after 10 min and became opaque after 15 min, and the reaction system became more and more opaque until completion of the polymerization. Samples were taken from the reaction mixture at different time to characterize the particle yield (*Y*_*p*_), number-average molecular weight (M_n_), and polydispersity index (PDI, M_w_/M_n_) of the PMS. All the PMS particles were spherical, and their size grew steadily with polymerization time (as shown in [Sec supplementary-material-1]). The changes of particle size and yield as a function of polymerization time are shown in Figure 1(a) and summarized in [Sec supplementary-material-1]. It can be seen clearly from Figure 1(a) and [Sec supplementary-material-1] that the particle diameter was 197 nm with *Y*_*p*_~2.36% after 10 minutes. With increasing reaction time, both the particle size and *Y*_*p*_ increased steadily. After 60 min, the particle size was 539 nm and *Y*_*p*_ reached up to 80%, which correspond to an 8% particle concentration (wt/v). Further measurement indicated that there was another ~2% PMS copolymer (wt/v) dissolved in solution, which was in good agreement with 10% (wt/v) monomer concentration.

In complementary experiments, we found that the PMS particles can be swollen neither in IA nor in liquid St, implying that copolymerization of MAH/St can only occur in solution rather than in the particles. This is a striking difference from conventional emulsion and dispersion polymerizations. These results suggest an unusual two-phase polymerization system; i.e., the polymerization mainly proceeds in solution, while the formation and growth of solid particles rely on the polymer chains formed in solution. This growth model for 2SP process was supported by an excellent correlation between the increase in particle size and yield shown in Figure 1(a).

Figure 1(b) depicts M_n_ and M_w_/M_n_ of PMS in the particles as a function of reaction time. It is obvious from Figure 1(b) that M_n_ of the PMS decreased dramatically from 240 to 90 kDa while M_w_/M_n_ increased from 5.3 to 7.5 with reaction time prolonging. This behavior is in contrast to that of conventional emulsion and dispersion polymerization, during which the molecular weight of the polymer product generally* increases* with reaction time. This result also differs from that of conventional solution polymerization, since the decrease in M_n_ is too steep. The two-phase nature of the reaction system can account for this unusual behavior. In this two-phase reaction system, the polymer chains formed in solution can aggregate to form the nuclei, and the newly formed polymer chains are then gradually adsorbed onto the existing nuclei. This would lead to lower solution viscosity, combined with the rapid decrease in monomer concentration, resulting in dramatic decrease in M_n_ and increase in M_w_/M_n_ of the PMS copolymer.

An important question about the 2SP polymerization is how can these PMS particles be formed and stably grow without aggregation/sedimentation/floatation, especially in the absence of any emulsifiers or stabilizers? It is well-known that various specific ligands are utilized during the fabrication of metal and inorganic nanoparticles in solution. Because the densities of metal and inorganic materials are much higher than that of solvents, normally nanosized colloids could only be stable in solution with the help of specific ligands, which are responsible for isolating particles and stabilizing them in reaction media. Since the densities of the polymers and the reaction media are quite close, the polymeric particles can be buoyant in media. Therefore, the main issue is how to prevent the polymer particles from coalescence.

In conventional emulsion and dispersion polymerization systems, the driving forces for segregation and stability of the polymer particles result from the repulsive interaction between charged surfaces of these particles or between solvated polymer chains. For the present 2SP polymerization system without any stabilizers, it is obvious that reaction medium would play an essential role. In order to clarify this point, we conducted alternating copolymerization of St/MAH in twelve different solvents and the results are summarized in [Sec supplementary-material-1]. It can be seen from [Sec supplementary-material-1] that, to get stable colloid, the solubility parameter of the solvent *δ*_S_ should be less than the solubility parameter of the PMS polymer (*δ*_PMS_ = 20.5 MPa^1/2^), and in particular Δ*δ* = *δ*_PMS_ − *δ*_S_ should be in the range of 1.8~4.5 MPa^1/2^. When Δ*δ* < 1.8 MPa^1/2^, a standard radical solution polymerization was observed, and a polymerization with immediate precipitation occurred when Δ*δ* > 4.5 MPa^1/2^. Based on the solubility parameters, it is evident that the interaction between the polymer chains is more favorable than that with the solvent. In other words, there is a tendency for the polymer chains to associate, but the interactions are not sufficiently strong to cause a precipitation. There is dynamics equilibrium between the soluble and aggregated polymer chains. As the local concentration of the polymer chains increased, the aggregates grew gradually and became sufficiently large, and finally nuclei were successfully formed.

During the process of nucleation and the following particle growth (deposition of the newly formed polymer chains onto the nuclei or particles), polymer chains inevitably undergo a desolvation process involving the release of solvent molecules, which was similar to crystallization of small molecule in solution. The desolvation process is a hardening process of the nuclei or the surface of the growing particles. The glass transition temperature (T_g_) of PMS was in the range of 126~160°C depending on its molecular weight. This high T_g_ value combined with the fact that PMS particles can be swollen neither in IA nor in liquid St, making us believe that the “dynamic hardening” prevents the conglutination of polymer particles during their growth. Moreover, we found that the PMS powder obtained after centrifugation and drying under vacuum showed morphology of isolated uniform spherical particles, and these dried particles could be easily redispersed in isoamyl acetate after ultrasonication. These results provide further evidence to support the mechanism above.

Furthermore, since this “dynamic hardening” process is short of strong driving force for segregation and stability, the particle formation process can be disturbed by agitation, and polymer particles with broad particle size distribution, coagulation, or even sedimentation were formed in the reaction system with stirring ([Sec supplementary-material-1]). The reason might be that agitation produced shear force that can disrupt the aggregation and adsorption of the polymer chains, and as a result the nucleation process was influenced and the stability of the reaction system was lost.

Based on these results, the key conditions for realizing a 2SP polymerization are the choice of appropriate reaction medium and the absence of agitation. The difference in solubility parameter of the polymer and the solvent should be in a suitable range, ensuring that the polymer chains are insoluble in the media (precipitation polymerization).

### 2.2. Extending 2SP Polymerization Strategy to a Wide Range of Common Olefins

Monoolefins (ethylene, propylene, and isobutene), di-olefins (butadiene, isoprene), and aromatic olefins (styrene and its derivatives), which are obtained by refining or cracking of hydrocarbons, are the primary monomers for the production of numerous plastic materials, synthetic fibers, and rubbers [28, 29]. It is well-known that a large amount of C4-C9 fractions are produced as by-products along with ethylene and propylene [30]. For example, in the steam cracking process, there are ~17% C4, ~16% C5, and 15% C9 fractions for each ton of ethylene. These fractions contain large amount of olefinic compounds with one or two double bonds; e.g., the C4 fraction contains 83% butadiene, isobutene, and 1-butene; the C5 fraction contains 53% isoprene, 1,4-pentadiene, and 1,3-pentadiene; and the C9 fraction contains ~44% dicyclopentadiene, indene, styrene, and their derivatives. Currently, the global ethylene output is ~170 million tons per annum, and it implies that there are over 50 million tons of olefinic compounds in C4, C5, and C9 fractions [28]. Therefore, it is of great significance to develop a simple strategy to transform these unused olefinic compounds into valuable polymeric materials.

On the basis of the above-mentioned background, we extended the 2SP polymerization strategy to a range of common olefinic monomers, including monoolefins, di-olefins, and aromatic olefins in C4-C9 fractions. The research work included two parts: Firstly, the 2SP copolymerization of single typical olefinic compound in C4/C5/C9 fractions with MAH and, secondly, 2SP copolymerization of complex C5 and C9 fractions with MAH.

As shown in Table 1, for 1-butene (monoolefin) and butadiene (di-olefin) in C4 fraction, their copolymerization with MAH in IA leads to a stable dispersion with 60% and 70% particle yield, respectively. For olefinic monomers in C5 fraction, the highest particle yield of 75% was obtained with isoprene in IA, and the particle yield was in the range of 60-70% for 1-pentene, cyclopentene, pentadiene, and 2-methyl-1-butene in IA/n-hexane (3/1) mixture solvent, while the particle yield of 2-methyl-2-butene and cyclopentadiene was only 40%, which is much lower than those of other monomers in C5 fraction. Three typical olefinic monomers, namely, *α*-methyl styrene, dicyclopentadiene, and indene in C9 fraction, all showed high reactivity to copolymerize with MAH, and the particle yields were all above 60%, while the highest particle yield reached up to 94.5% for indene in IA/n-heptane (2/1). Exceptional situations come from 2-butene and 2-pentene and the reaction system remains clear solution with zero particle yields.

**Figure 2:**
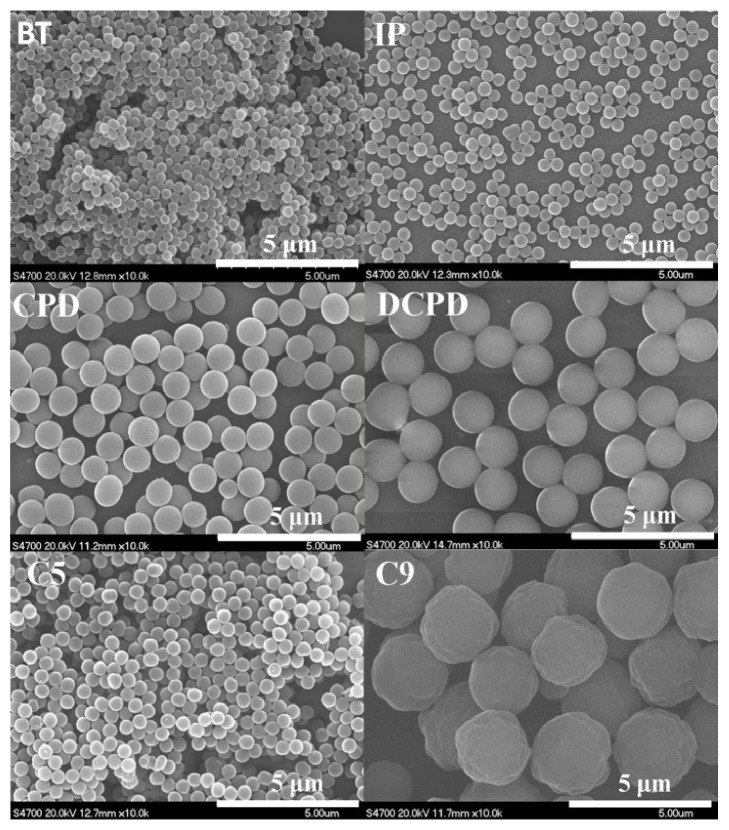
**Characterization of the polymer particles produced by 2SP polymerization.** SEM images of the nanoparticles produced by 2SP polymerization: 1-butene (BT), isoprene (IP), cyclopentadiene (CPD), dicyclopentadiene (DCPD), and C5 and C9 fractions.

On the whole, in a single or mixed solvent almost all of these olefins could copolymerize with MAH to produce alternative copolymers with the reactivity of aromatic olefin > conjugating olefin > *α*-olefin, while the olefins like 2-butene and 2-pentene have no reactivity for copolymerization with MAH. It is worth noting that although the *α*-olefinic monomers generally have low reactivity for radical homopolymerization, they have relatively high reactivity for radical copolymerization, particularly with MAH and its derivatives. Furthermore, the copolymerization of these olefins with MAH not only could form stable milky dispersions in different solvent but also always led to particles of uniform size, regardless of the nature of the olefinic monomers, as shown in Figure 2. The FT-IR spectrum of the poly(maleic anhydride-alt-1-butene) is provided in the supporting information [Sec supplementary-material-1] to confirm the incorporation of olefinic monomers into the polymer backbones.

**Figure 3:**
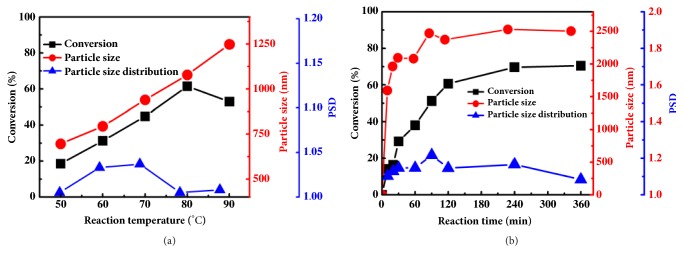
**Polymerization results of C5 and C9 fractions with MAH. (a)** Monomer conversion, particle diameter, and PSD of C5 fractions/MAH at different polymerization temperatures. Reaction condition: the total concentration of olefines and MAH is 0.5 mol/L, in IA/n-hexane (3/1) with 5 wt% AIBN as initiator, reaction time 6 hours.** (b)** The variation of particle yield, particle diameter, and PSD of C9 fractions/MAH as a function of polymerization time. Reaction condition: total monomers concentration is 33 wt% in xylene, at 65°C with 2.5 wt% AIBN as initiator.

Following that, we investigated the 2SP polymerization behavior of MAH with industrial C5 and C9 fractions (directly as raw materials with main compositions shown in Tables [Sec supplementary-material-1] and [Sec supplementary-material-1]), and the polymerization results are shown in Figure 3.

Figure 3(a) demonstrates the variation of monomer conversion, particle size, and particle size distribution (PSD) as a function of temperature for 2SP polymerization of C5 fraction with MAH. It can be clearly seen that, with temperature increasing, the particle size steadily increased from 696 nm to 1248 nm with narrow PSD (less than 1.05), and the monomer conversion reached a maximum value of 61.5% at 80°C. This one-step conversion of total olefinic compounds in C5 fraction is quite high, which is very meaningful from an industrial viewpoint. As we all know, routine C5 fraction contains more than 30 components, and there are about 48% dienes, and another 12% olefines as depicted in [Sec supplementary-material-1]. Besides, there are 0.98% alkynes that are well-known as inhibitor for radical polymerization due to high chain transfer constant and extremely low reactivity of the resultant macroradicals. The polymerization results demonstrate that the impurities in C5 fraction show no obvious adverse effect on the 2SP polymerization process due to the high copolymerization reactivity of olefinic compounds with MAH. Furthermore, a variety of olefines can be successfully incorporated into one molecular chain through alternating copolymerization, leading to the formation of multicomponent copolymer based on C5 and MAH.

Figure 3(b) depicts the variation of monomer conversion, particle diameter, and PSD as a function of reaction time for 2SP polymerization of C9 fraction with MAH. It is obvious from Figure 3(b) that both the monomer conversion and the particle size increased steadily with polymerization time, demonstrating the living growth property of 2SP polymerization process. The one-step conversion of olefinic compounds in C9 fraction reached up to 71% and the diameter of the obtained C9-MAH particles was in the range of 1600-2500 nm. This high one-step conversion could be rational based on the composition of C9 fraction. Even though the C9 fraction is more complex and has more than 150 components, the olefinic compounds in C9 fraction have higher reactivity to copolymerize with MAH, as shown in [Sec supplementary-material-1]. As a result, the polymerization rate is fast and the one-step conversion of olefinic compounds is high. In addition, the monomer concentration is up to 33 wt%, leading to C9-MAH particles with much bigger size. It is worth pointing out that the growth rate of particle size became much lower when the particle diameter was big. Consequently, the PSD of C9-MAH particles decreased gradually to near unity, indicating the self-limiting aspect of 2SP polymerization process.

In brief, the experimental results confirm the universality of the 2SP polymerization process. Furthermore, due to the high copolymerization reactivity of olefinic compounds with MAH, functional copolymer NPs with narrow PSD and complex composition can be successfully obtained through 2SP copolymerization of MAH and C5/C9 fractions without any purification process. More importantly, the as-formed functional particles can be easily separated from the reaction system with high yield.

## 3. Discussion

In our previous work, we reported the preparation of uniform maleic anhydride/vinyl acetate copolymer particles with alkyl esters as the reaction medium in the absence of any stabilizers [31–33]. Because there were no stabilizers in the reaction system and the particle growth behavior was similar to that of dispersion polymerization, we named this facile method “stabilizer-free dispersion polymerization.” To get deep insight into the mechanism of this novel polymerization system, further investigation was carried out. The experimental results demonstrated that there were obvious differences in locus of polymerization, polymerization kinetics, and mechanic feature between this novel polymerization system and conventional dispersion polymerization system. Therefore, it is meaningful to study the particle formation process and clarify the mechanism of this facile polymerization process. In the present paper, we developed a facile and efficient 2SP polymerization based on our previous work. The polymerization of a variety of monomers was carried out via 2SP polymerization and the particle formation mechanism was investigated in detail.

### 3.1. Particle Formation Mechanism in 2SP Polymerization

**Figure 4:**
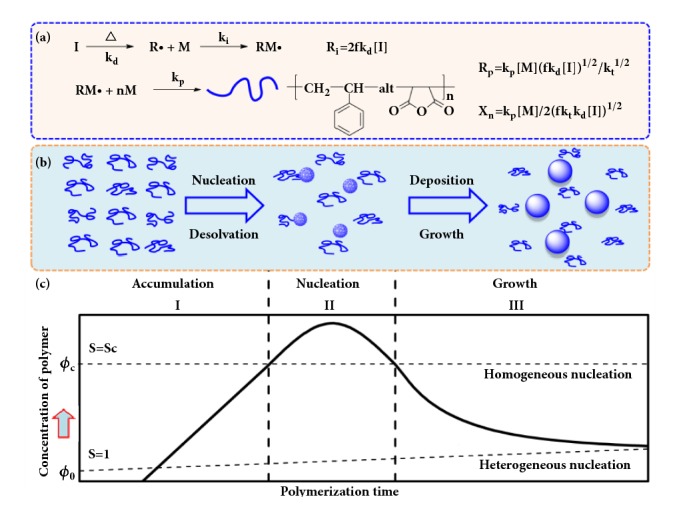
**Nucleation and particle growth mechanism for 2SP polymerization. (a)** The polymerization kinetic equations of St/MAH.** (b)** Schematic illustration for the nucleation and growth process of the PMS particles.** (c)** A classic LaMer diagram plot of the 2SP polymerization of St/MAH.

As illustrated in the previous section, the polymerization behavior and particle stabilization mechanism of the 2SP polymerization process are significantly different from that of conventional emulsion/suspension/dispersion polymerization process [34]. Therefore, referring to the solution crystallization theory, we proposed a novel mechanism to interpret the nucleation process and growth feature of the particles during 2SP polymerization based on thermodynamic analysis, and the schematic illustration is shown in Figure 4.

#### 3.1.1. Polymerization in Solution

Initially, the reaction system consisted of monomers (St/MAH), solvent (IA), and initiator (AIBN). The thermal decomposition of AIBN leads to the formation of primary radicals and monomer radicals, which initiated a standard radical alternating copolymerization of St and MAH. Based on the two-phase nature of 2SP polymerization, the main function of alternating copolymerization in solution is to supply PMS copolymer building-blocks for nucleation and particle growth. Thus, we can just focus on the amount and nature of the as-formed PMS chains rather than polymerization mechanism involved.

As shown in Figure 4(a), the chain number of the PMS building-blocks is generated at a rate of *R*_*i*_ = 2fk_d_I and the mass of the PMS building-blocks is produced at a rate of *R*_*p*_ = k_p_[M] (fk_d_[I])^1/2^/k_t_^1/2^, while the chain length or M_n_ can be determined by *X*_*n*_ = k_p_[M]/2(fk_t_k_d_[I])^1/2^. With the polymerization proceeding, the concentration of monomers and initiator will decrease gradually so that *R*_*p*_ and *X*_*n*_ would decrease gradually. This deduction from Figure 4(a) is in good agreement with the experimental results shown in Figure 1(b) and [Sec supplementary-material-1].

#### 3.1.2. Nucleation

According to the phase-separation theory of Flory-Huggins [35], when temperature, pressure, and solvent are fixed, the chemical potential of solvent in a polymer solution can be described as

(1) $$\begin{array}{c} \Delta \mu_{1}=RT\left[\;\ln\phi_{1}+\left(\;1-\frac{1}{x}\;\right)\phi_{2}+\chi \phi_{2}^{2}\;\right] \end{array}$$

where* ϕ*_*1*_ is volume fraction of the solvent,* ϕ*_*2*_ is volume fraction of the polymer, x is the degree of polymerization of the polymer, and* χ* is the Flory-Huggins interaction parameter (depending on the chemical structure of the polymer and the solvent). As temperature, pressure, solvent, and* χ* were fixed, the critical condition under which phase-separation took place can be calculated based on (1), and the critical value of* ϕ*_*2*_ is described as

(2) $$\begin{array}{c} \phi_{2c}=\frac{1}{\sqrt{x}} \end{array}$$

Phase-separation will take place in reaction medium when the volume fraction of the polymer* ϕ*_*2*_ reached the critical value. Obviously,* ϕ*_*2c*_ is inversely proportional to x; that is, the higher x, the smaller* ϕ*_*2c*_.

In view of the classic theory of nucleation and growth, the nucleation and growth process of the 2SP polymerization can be described briefly below (as shown in Figure 4(b)) [10, 21]. As polymerization proceeding, the concentration of PMS “building-blocks” constantly increased. The accumulated concentration of PMS in IA gradually increased and exceeded over a critical point *ϕ*_c_ (about 2 wt %), above which all PMS produced in solution would become consuming materials solely for nucleation and growth of particles. Since *R*_*p*_ (*R*_*p*_= k_p_[M] (fk_d_[I])^1/2^/k_t_^1/2^) is the molar mass of PMS produced in unit time, the volume fraction of the as-formed polymer* ϕ* can be calculated based on *R*_*p*_. In light of the formation process of nanocrystal, supersaturation could be considered as the driving force for nucleation and growth of the polymer particles. The level of supersaturation,* S*, can be defined as* ϕ*/*ϕ*_*0*_, where* ϕ*_*0*_ is the equilibrium polymer concentration in the polymer solution. Noting that *R*_*p*_ and *X*_*n*_ would steadily decrease due to the decrease of [M] and [I] with polymerization time, thus a classic LaMer diagram plot was formed, as shown in Figure 4(c) [36].

It can be clearly seen from Figure 4(c) that when the concentration of PMS increased to a value over* ϕ*_*c*_, PMS polymer chains, particularly polymer chains with higher molecular weight (higher x), would overcome the kinetic barrier and aggregate to form nuclei in a homogeneous solution (homogeneous nucleation) [37]. Hence, the molecular weight of the PMS particles is relatively high in the early stage and decreased gradually as polymerization proceeding, as shown in Figure 1(b).

The Gibbs free energy change of the formation of spherical nucleus with radius r from the solution is expressed as

(3) $$\begin{array}{c} \Delta G=4\pi r^{2}\gamma +\frac{4}{3}\pi r^{3}\Delta G_{V} \end{array}$$

where *γ* is the surface free energy per unit area and* △G*_*v*_ is the free energy per unit volume of a polymer particle [38].* △G*_*v*_ can be expressed as the change between the free energy of the PMS in particle phase and in solution and* △G*_*v*_ as a function of supersaturation *S* is given in

(4) $$\begin{array}{c} \Delta G_{V}=-\frac{RT\ln S}{V_{m}} \end{array}$$

where V_m_ is the molar volume of the PMS in polymer particle.

According to the classical theory of nucleation, some of the newly formed nuclei, which are smaller and not thoroughly desolvated, are less stable and cannot grow further but only dissolve back into the solution. Based on (3) and (4), it is possible to get the critical radius by differentiating* ΔG* with respect to r and setting it to zero, d*ΔG/dr *= 0.

(5) $$\begin{array}{c} r_{c}=\frac{-2\gamma }{\Delta G_{V}}=\frac{2\gamma V_{m}}{RT\ln S} \end{array}$$

A maximum free energy is obtained at *r*_*c*_, and the as-formed nucleus will pass through this point to form a stable nucleus. In other words, *r*_*c*_ is the minimum size of the nuclei that can resist dissolution and grow further. The critical free energy* △G*_*c*_ can be obtained by substituting (5) into (3), which is the free energy necessary to form a stable nucleus [see (6)]. If the increase rate of the particles number *N* is defined as the rate of nucleation, it can be written in the Arrhenius form in terms of* △G*_*c*_ [see (7)].

(6) ΔGc=16πγ33ΔGV2=16πγ3Vm23RTln⁡S2

(7) dNdt=Aexp⁡−ΔGckT=Aexp⁡16πγ3Vm23RTln⁡S2

As can be seen in Figure 4(c), the degree of supersaturation *S* is high enough to overcome the energy barrier for nucleation in stage II; thus nucleation occurs in the reaction system, resulting in the formation and accumulation of stable nuclei. Since the concentration of monomers and initiator decreased gradually with polymerization time, the rate of polymerization in solution (production rate of PMS chains) would decrease accordingly, and* ϕ*_*2*_ (or* S*) would decrease accordingly. When the value of *S* was *S*_*c*_ again, the nucleation rate became zero (there were no new nuclei formed). Below this level, the system entered the growth stage (stage III), during which nucleation was effectively stopped and the particles kept growing as long as the polymerization proceeded.

#### 3.1.3. Particle Growth

The growth of the particles due to the deposition of the polymer chains onto the particles is governed by the diffusion and adsorption of the newly formed PMS chains on the particle surface until the end of the polymerization process. In fact, the mechanistic study on the formation of nanocrystals indicated that the burst of nucleation enabled separation of the nucleation and the subsequent diffusion-controlled growth process, leading to the formation of monodisperse nanocrystals [39]. Similarly, the growth process of the particles in 2SP polymerization could be quantitatively described as follows. At some transient time t, the growth rate of the spherical particles depended solely on the PMS chains adsorbed onto the particles (i.e., *R*_*p*_). In this case, the relationship between *R*_*p*_ and the rate of the particle volume change is given by

(8) $$\begin{array}{c} J=R_{P}=\frac{4\pi r^{2}}{V_{m}}\frac{dr}{dt} \end{array}$$

When the polymer diffusion is the rate-determining step for the particle growth, the flux of the polymers onto the surface of the particles can be calculated as

(9) $$\begin{array}{c} J=4\pi rD\left(\;C_{bulk}-C_{s}\;\right) \end{array}$$

where D is the diffusion constant and C_bulk_ and C_s_ are the concentration of PMS in solution and at the surface of the polymer particle.

From (8) and (9), an expression for* dr/dt* is obtained:

(10) $$\begin{array}{c} \frac{dr}{dt}=\frac{V_{m}D}{r}\left(\;C_{bulk}-C_{s}\;\right)=\frac{R_{p}V_{m}}{4\pi r^{2}} \end{array}$$

It is obvious that the bigger the* r*, the smaller the* dr/dt*. Furthermore, *R*_*p*_ would decrease with polymerization proceeding due to the decrease of [M] and [I]. Hence,* dr/dt* would decrease more significantly with the increase of* r*, resulting in a self-regulating mechanism of the size distribution during the particle growth process. In other words, the smaller the particle, the faster the particle growth; on the contrary, the big particle grew much more slowly. Consequently, the size of the particles in the reaction system tends to be uniform, and nearly monodisperse particles can be successfully obtained.

For 2SP polymerization of St/MAH, the particle yield, particle size, and M_n_ of PMS copolymer at different polymerization time are summarized in [Sec supplementary-material-1]. Assuming that the PMS particles are monodisperse with spherical shape and on the basis of *Y*_*pt*_, *D*_*t*_, and *M*_*nt*_ shown in [Sec supplementary-material-1], the average number of particles per unit volume *N*_*t*_ and the average number of PMS chains per particle *ξ*_*t*_ can be obtained by the following equation:

(11) Nt=6WYptπρDt3

(12) ξt=WYptNANtMnt=πρNADt36Mnt

where W is the weight of monomers per unit volume of solution; *ρ* is the density of the PMS copolymer; and *N*_A_ is Avogadro's number; *Y*_*pt*_, *D*_*t*_, and *M*_*nt*_ are the yield, diameter, and M_n_ of the PMS particles at different reaction time* t*.

**Figure 5:**
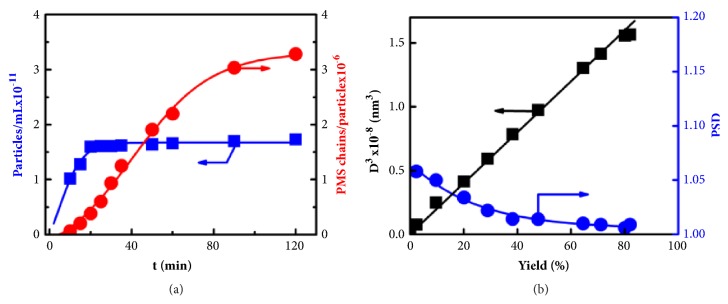
**Self-limiting and living growth aspect of 2SP polymerization. (a)** The variation of number of PMS particles per unit volume N_t_ and number of PMS chains in a particle *ξ*_t_ as a function of polymerization time.** (b)** The variation of cube of PMS particle diameter and particle size distribution as a function of particle yield.

Figure 5(a) shows the variation of *N*_*t*_ and *ξ*_*t*_ as a function of polymerization time. It is obvious that the number of particles increased sharply in the early stage of polymerization and then remained essentially constant. This result indicates that the homogeneous nucleation of the polymer particles occurred only in the beginning of the polymerization (about 20 min). When the yield of the polymer particles increased up to 20 wt% (i.e., t=20 min in Figure 5(a)), the nucleation process is complete. On the other hand, the number of polymer chains within each particle continually increased, indicating that particle growth occurred heterogeneously through surface-deposition of the newly formed polymer chains. These experimental results were in good agreement with the deduction on the basis of Figure 4(c).

To get better understanding of the particle growth process, the volume and size distribution of the obtained PMS particles from [Sec supplementary-material-1] are plotted against particle yield. As shown in Figure 5(b), it is obvious that the particle volume increased linearly with particle yield. This result shows the “living growth” nature of the particles [40]. Furthermore, the size distribution of the PMS particles was very narrow (PSD below 1.05). The value of PSD gradually decreased to near unity with increasing particle yield, indicating the self-limiting particle size aspect of 2SP polymerization. As a result, nearly monodisperse particles were obtained in the end of the 2SP polymerization process.

### 3.2. Application of the 2SP Polymerization

As mentioned previously, the heterogeneous polymerization accounts for a large amount of polymer products, especially for polymeric particles, owing to its unique features, such as the ease of heat removal, facile separation of polymer product, and direct formation of solid polymer particles [1–6]. Generally, monodisperse nanoscopic and microscopic polymeric beads have found widespread applications in the fields of separation media, ion-exchange beads, toners, coatings, calibration standards, and medical diagnostics. For most of these applications, the control of particle size and size distribution are critical. In comparison with the conventional heterogeneous polymerization approaches [13–18], the 2SP polymerization process is much simpler and more efficient. There is not any stabilizer in the reaction system; consequently, pure polymeric materials are obtained without the need of further purification. Most importantly, the as-formed uniform solid particles can be easily separated from the reaction media through centrifugation or filtration, and the supernatant can be reused for subsequent polymerization. Based on the above discussion, we may reasonably get a conclusion that 2SP polymerization is the most simple and efficient strategy to produce uniform polymeric NPs.

Currently, the global output of C4, C5, and C9 fractions is over 100 million tons, and there are huge amounts of olefinic compounds in these fractions. Due to the technical difficulty and high cost for separation and purification, the utilization level of these olefinic monomers is very low. Apart from a small part being used as raw material for petroleum resin (two million tons), most of them are merely used as a low-cost fuel (over fifty million tons). Thus, it is of great urgency to develop a novel strategy to convert these complex C4-C9 fractions into valuable polymer materials.

Despite extremely low reactivity for radical homopolymerization, our experimental results demonstrated that almost all of these olefins could copolymerize with MAH through 2SP polymerization, to form copolymer particles with high yield (Table 1). Especially, even for the industrial C5 and C9 fractions of highly complex compositions, copolymer NPs with narrow size distribution could be successfully formed with reasonable one-step conversion (Figure 3). Most importantly, the polymeric NPs can be easily separated from the reaction medium and the supernatant liquid can be reused, making this strategy a truly “green” polymerization process. Moreover, compared with the currently produced nonpolar olefinic polymers and copolymers, the as-formed copolymers containing reactive/polar-functional groups could be directly used as polar-functional polymeric material with high T_g_ or transformed into water soluble copolymers, which have a higher added value. Due to the above-mentioned advantages, the 2SP polymerization of MAH and C5/C9 fractions can be scaled to an industrial level, paving new pathway for the efficient utilization of C5/C9 fractions.

## 4. Conclusions

In this work, we developed a novel heterogeneous polymerization strategy, beyond emulsion/dispersion/suspension, termed as 2SP polymerization, in which polymer NPs of well-defined size were generated through the alternating copolymerization of olefinic compounds with MAH or its derivatives in the absence of any stabilizers or crosslinker. A possible mechanism was proposed for this 2SP polymerization process: nucleation of the particles occurred homogeneously only at the early stage of the polymerization, and the particle growth is mainly due to the surface-deposition of the newly formed polymer chains. It is worth noting that the choice of reaction medium is crucial for realizing a 2SP polymerization. Moreover, the 2SP polymerization strategy can be extended to a range of common olefinic monomers in C4, C5, and C9 fractions (with the exception of 2-butene and 2-pentene). This technique has led to two revolutionary advances: the generation of copolymers particles from single olefin or complex olefin mixtures (C4/C5/C9 fractions without separation) and the facile fabrication of functional polymeric NPs with narrow particle size distribution. Furthermore, the separation of the NPs from the reaction medium is simple and the supernatant liquid can be reused, making this strategy a green process, which has broad prospects for industrial applications. Hence, this novel synthetic strategy opens a new pathway for the efficient utilization of olefin mixtures and the as-formed functional, organic nanoparticles will impact a broad range of fields ranging from separations to biomedical tracers and devices.

## 5. Materials and Methods

### 5.1. Materials

St was purchased from Beijing Chemical Factory and distilled under reduced pressure to remove the inhibitor. Butadiene, 2-butene, 1-butene, and C9 fraction ([Sec supplementary-material-1]) were donated by Yanshan Co. (Beijing) of Sinopec; isoprene (analytical grade) and indene (analytical grade) were purchased from Alfa Aesar; 1-pentene (98%), 2-pentene (98%), 2-methyl-1-butene (99%), 2-methyl-2-butene (99%), 1,3-pentadiene (95%), cyclopentene (98%), and dicyclopentadiene (95%) were from J&K Chemical and 1,4-pentadiene (95%) was from TCI; cyclopentadiene was obtained from dicyclopentadiene by distillation decomposed at 200°C; C5 fraction ([Sec supplementary-material-1]) was donated by Yuhuang Co. (Shandong); and all of olefinic monomers were used without further purification. MAH was purchased from Acros and used as received. IA and all other solvents were of analytical grade from Beijing Chemical Factory and used as received. AIBN was purchased from Beijing Chemical Factory and recrystallized from ethanol. Benzoyl peroxide (BPO) bought from Beijing Chemical Factory was dissolved in chloroform and recrystallized from ethanol in ice-water bath.

### 5.2. Methods

#### 5.2.1. Typical Procedure for 2SP Polymerization

The apparatus for 2SP polymerization consists of a three-neck round-bottom flask equipped with a condenser and a gas inlet. MAH and olefinic monomer or mixture with a fixed concentration and molar feed ratio, initiator, and solvent were added to the three-neck round-bottom. After purging N_2_ for 30 min, the reactor was placed into a water bath at a fixed temperature. No agitation was used during polymerization. To get deep insight into the particle formation process, samples were taken from the reaction mixture at different time to characterize *Y*_*p*_, M_n_, and PDI of PMS.

To investigate the influence of monomer feed ratio and monomers concentration on the size, size distribution, and composition of the resultant polymer particles, the copolymerization of MAH and St was carried out in IA with a wide range of monomer feed ratios and monomers concentration. To evaluate the effect of solvent on the size and morphology of the particles, the copolymerization of St and MAH was carried in various solvents and mixed solvents. Typical olefinic compounds in C4/C5/C9 fractions and even industrial C5/C9 fractions were copolymerized with MAH to extend the scope of monomers for 2SP polymerization strategy.

### 5.3. Characterization

The yield of polymer particles at a certain reaction time t was determined by the following equation:

(13) $$\begin{array}{c} Y_{pt}=\frac{W_{pt}}{W_{m}}\times 100\% \end{array}$$

where *W*_*pt*_ is the weight of the polymer at certain reaction time and *W*_m_ is the weight of the added monomers.

The morphology of polymeric particles was observed by HITACHI S-4700 Scanning Electron Microscope (SEM). Particle sizes and size distributions were obtained from electron micrographs. Five hundred particles were measured at least and the average size of the polymer particles is calculated according to the following formula:

(14) Dn=∑i=1kniDi∑i=1kni;

(15) Dw=∑i=1kniDi4∑i=1kniDi3;

(16) PSD=DwDn

where *D*_n_ is the number-average diameter; *D*_w_ is the weight-average diameter; n_i_ and *D*_i_ are the number and the diameter of the particles, respectively; PSD is defined as the ratio of *D*_w_ to *D*_n_.

The chemical composition of the copolymer particles was measured by a ThermoNicolet Nexus 670 FT-IR spectrometer and a Bruker AV600 NMR spectrometer. The molar ratio of MAH in the copolymer is measured by a titration method. The molecular weight and its PDI of PMS copolymers were determined by a Waters GPC515-2410 system equipped with a Waters R410 refractive index detector and packing columns (Waters Styragel HT3-5-6E), using THF as an eluent at a flow rate of 1 mL/min, calibrated with polystyrene standard samples.

## Figures and Tables

**Table 1:** **Copolymerization results of olefins with MAH. **Copolymerization of typical olefins in C4/C5/C9 fractions with MAH^*※*^.

**Name**	**Observation**	**Yield**
**C4 fraction**		
Butadiene^a^	dispersion	70%
1-Butene^a^	dispersion	60%
2-Butene^a^	solution	0

**C5 fraction**		
1-Pentene^b^	dispersion	65%
Cyclopentene^b^	dispersion	60%
2-Methyl-1-butene^b^	dispersion	70%
2-Methyl-2-butene^b^	dispersion	40%
2-Pentene^b^	solution	0
Isoprene^a^	dispersion	75%
1,4-Pentadiene^b^	dispersion	70%
1,3-Pentadiene^b^	dispersion	65%
Cyclopentadiene^c^	dispersion	40%

**C9 fraction**		
*α*-Methyl styrene^a^	dispersion	60%
Dicyclopentadiene^c^	dispersion	65%
Indene^d^	dispersion	94.5%

^*※*^Olefin: MAH is 1:1 (mol), monomers concentration is 20 wt%, AIBN is 3 wt% relative to monomers, and reaction temperature is 70°C for 6 hours.

^a^IA.  ^b^IA/n-hexane (3/1).  ^c^ Ethyl oenanthate/n-hexane (1/1), AIBN 5 wt%.  ^d^IA/n-heptane (2/1).

## Supplementary Materials

Supplementary Materials
** Figure S1:** SEM images and FT-IR/^13^C NMR spectra of copolymer microspheres at different monomer feed ratios and concentrations. The molar ratio of MAH to St is (A) 4:1, (B) 3:1, (C) 2:1, (D) 1:2, (E) 1:3, (F) 1:4, (G) 1:1, (H) FT-IR, and (I) ^13^C NMR spectra of polymer. (A~C) the concentration of St was fixed at 1.0 mol/L, and the concentration of MAH varied from 4 mol/L to l mol/L; (D~G) the concentration of MAH was fixed at 1 mol/L. The reaction media were IA, and the reaction temperature was 70 ± 1°C.** Figure S2:** SEM images of copolymer microspheres at different reaction time: (A) 10 min; (B) 15 min; (C) 20 min; (D) 25 min; (E) 30 min; (F) 35 min; (G) 50 min; (H) 60 min; (I) 90 min. The scale bar is 5 *μ*m.** Figure S3: **(A) SEM of PMS particles prepared with stirring (top), reaction condition: MAH (2.452 g), St (2.60 g), and AIBN (0.0329 g), reaction temperature 70 ± 1°C. TEM micrographs of the PMV copolymer microspheres prepared with stirring, reaction time: (B) 3 hours; (C) 4 hours. Preparation conditions: [MAn] = [VAc] = 1.0 M; BPO 0.8 wt% relative to monomers; temperature, 80 ± 1°C.** Figure S4:** the FT-IR spectrum of the poly(maleic anhydride-alt-1-butene).** Table S1: **the particle size, particle size distribution, and composition of microspheres formed with different molar feed ratio of MAH/St and monomers concentrations.** Table S2:** some parameters of PMS particles obtained at different polymerization time.** Table S3:** effect of solvents on alternative copolymerization of St and MAH.** Table S4:** The composition of C_5_ mixture.** Table S5: **the composition of C9 fraction.
