# Supplementary material for: Self-Stabilized Precipitation Polymerization and Its Application
Source: Research (Wash D C). 2018 Sep 10;2018:9370490. doi: 10.1155/2018/9370490 (PMC6750106; doi:10.1155/2018/9370490)
Supplement: Supplementary Materials — Figure S1: SEM images and FT-IR/13C NMR spectra of copolymer microspheres at different monomer feed ratios and concentrations. The molar ratio of MAH to St is (A) 4:1, (B) 3:1, (C) 2:1, (D) 1:2, (E) 1:3, (F) 1:4, (G) 1:1, (H) FT-IR, and (I) 13C NMR spectra of polymer. (A~C) the concentration of St was fixed at 1.0 mol/L, and the concentration of MAH varied from 4 mol/L to l mol/L; (D~G) the concentration of MAH was fixed at 1 mol/L. The reaction media were IA, and the reaction temperature was 70 ± 1°C. Figure S2: SEM images of copolymer microspheres at different reaction time: (A) 10 min; (B) 15 min; (C) 20 min; (D) 25 min; (E) 30 min; (F) 35 min; (G) 50 min; (H) 60 min; (I) 90 min. The scale bar is 5 μm. Figure S3: (A) SEM of PMS particles prepared with stirring (top), reaction condition: MAH (2.452 g), St (2.60 g), and AIBN (0.0329 g), reaction temperature 70 ± 1°C. TEM micrographs of the PMV copolymer microspheres prepared with stirring, reaction time: (B) 3 hours; (C) 4 hours. Preparation conditions: [MAn] = [VAc] = 1.0 M; BPO 0.8 wt% relative to monomers; temperature, 80 ± 1°C. Figure S4: the FT-IR spectrum of the poly(maleic anhydride-alt-1-butene). Table S1: the particle size, particle size distribution, and composition of microspheres formed with different molar feed ratio of MAH/St and monomers concentrations. Table S2: some parameters of PMS particles obtained at different polymerization time. Table S3: effect of solvents on alternative copolymerization of St and MAH. Table S4: The composition of C5 mixture. Table S5: the composition of C9 fraction. [file 9370490.f1.docx]

*Supplementary Materials*

**Self-stabilized precipitation polymerization and its application**

Zhenjie Liu^1^**^#^**, Dong Chen^1^**^#^**, Jinfang Zhang^1^, Haodong Liao^1^, Yanzhao Chen^1^, Yingfa Sun^1^, Jianyuan Deng^1^ Wantai Yang^1,2^*

^1^College of Materials Science and Engineering, Beijing University of Chemical Technology, Beijing, 100029, China.

^2^State Key Laboratory of Chemical Resource Engineering, Beijing University of Chemical Technology, Beijing, 100029, China

*Correspondence to: [yangwt@mail.buct.edu.cn](mailto:yangwt@mail.buct.edu.cn)

^#^These authors contributed equally to this work.

**
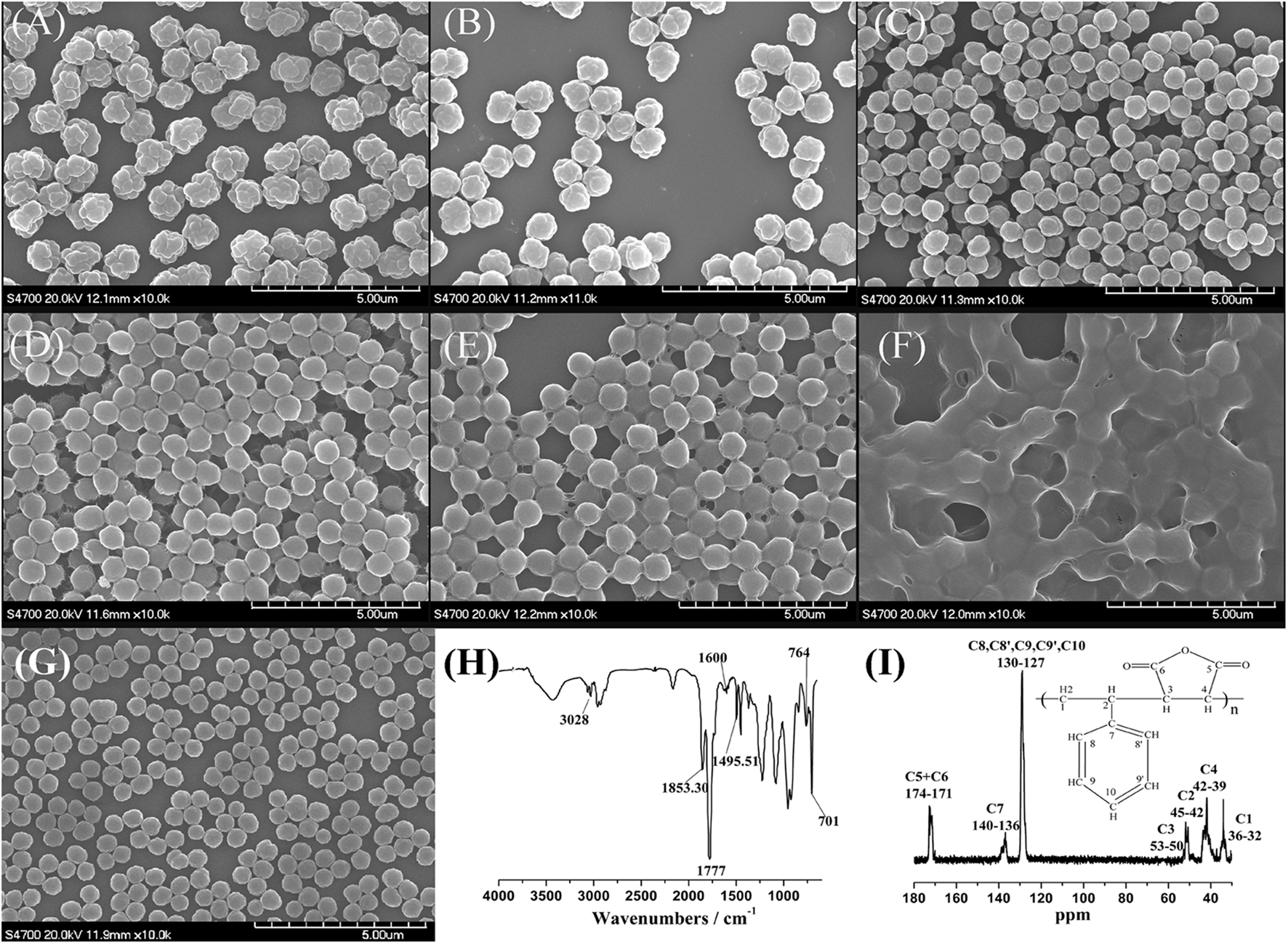
**

**Figure S1.** SEM images and FT-IR/^13^C NMR spectra of copolymer microspheres at different monomer feed ratios and concentrations. The molar ratio of MAH to St is: (A) 4:1, (B) 3:1, (C) 2:1, (D) 1:2, (E) 1:3, (F) 1:4, (G) 1:1. (H) FT-IR and (I) ^13^C NMR spectra of polymer. (A~C) the concentration of St was fixed at 1.0 mol/L, and the concentration of MAH varied from 4 mol/L to l mol/L; (D~G) the concentration of MAH was fixed at 1 mol/L. The reaction media was IA, and the reaction temperature was 70±1°C.

**Polymerization process of MAH and St and the formation process of PMS particles.**

MAH (7.356 g), St (7.8 g), IA (135 mL) and AIBN (0.0987 g) were added into the experimental apparatus respectively. After purging N_2_ for 30 min, the reactor was placed into a water bath at 70±1°C. No agitation was used during polymerization. At different reaction time, 2 mL reaction solution was drawn from the reaction system and poured into 5 mL cool IA to stop the reaction, and then the resultant solution was centrifugated at a rate of 4000 rad/min within 20 min and the supernatant was removed. The particles were re-suspended in a certain solvent by ultrasonication within 30 min before being characterized.


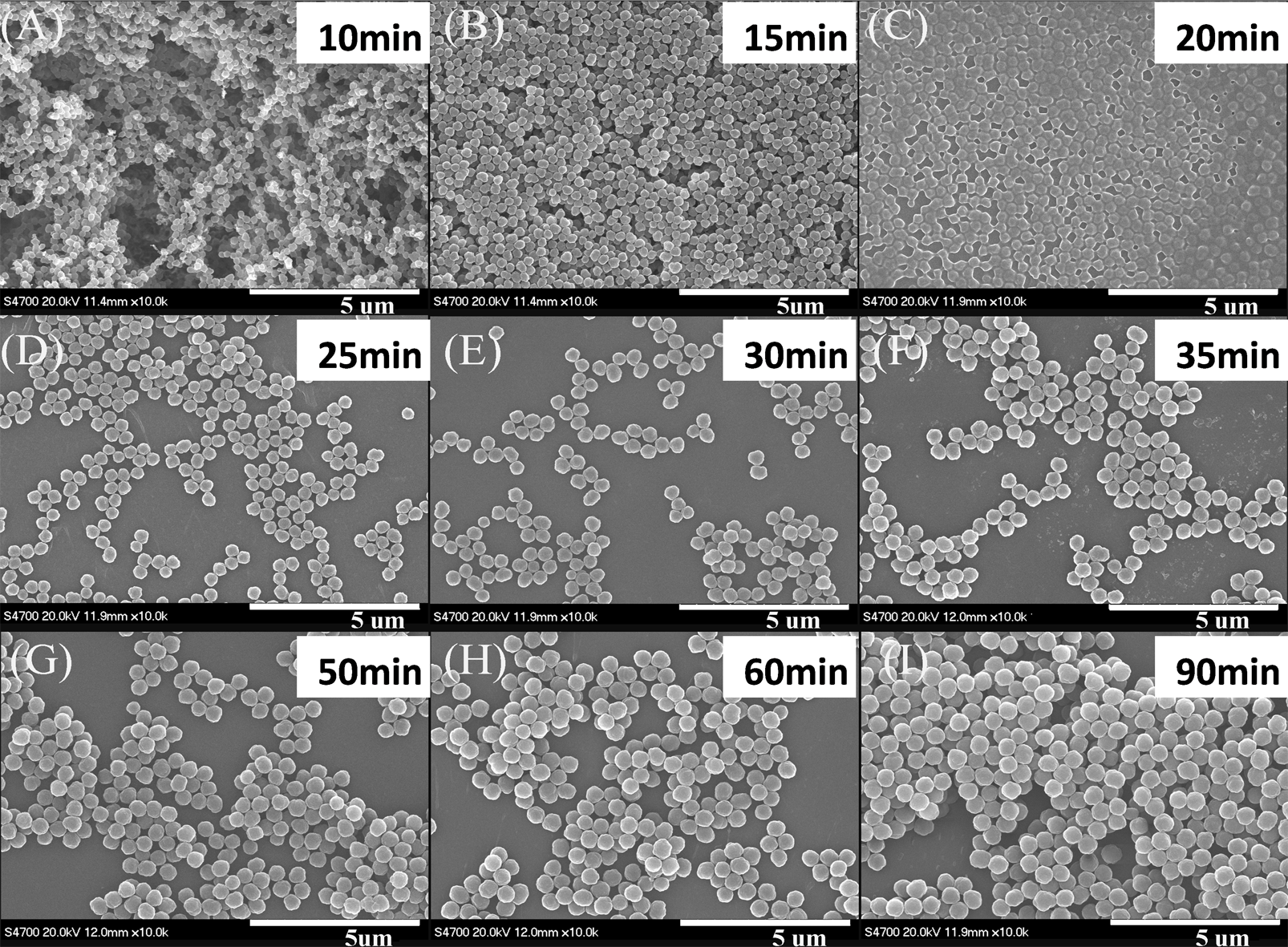


**Figure S2.** SEM images of copolymer microspheres at different reaction time: (A) 10 min; (B) 15 min; (C) 20 min; (D) 25 min; (E) 30 min; (F) 35 min; (G) 50 min; (H) 60 min; (I) 90 min. The scale bar is 5 μm.


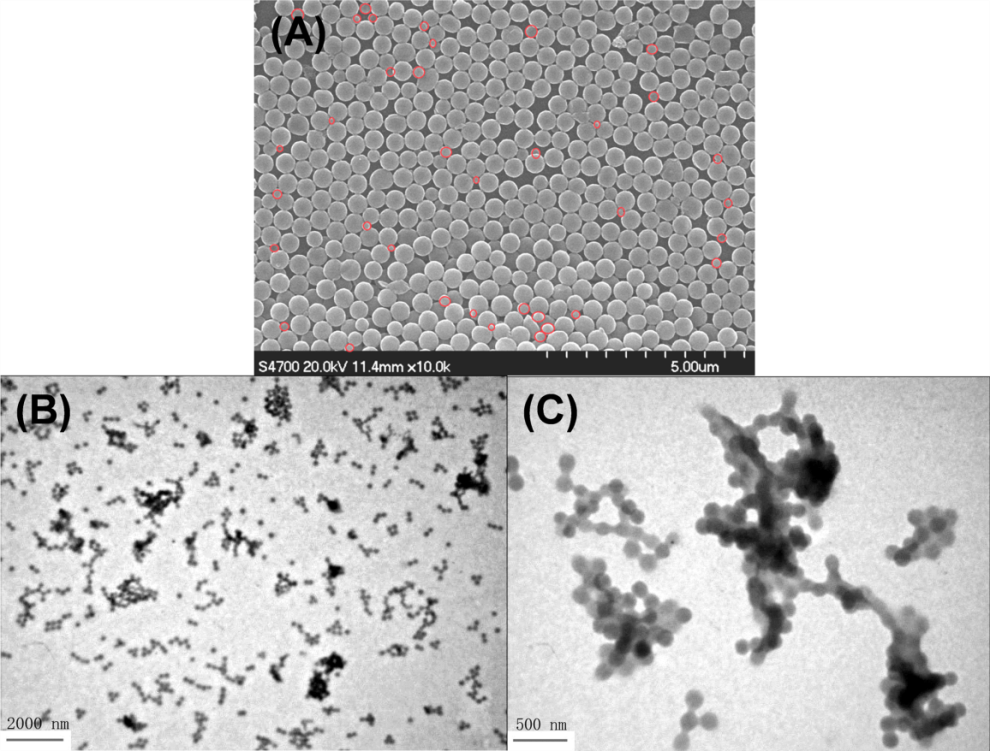


**Figure S3.** (A) SEM of PMS particles prepared with stirring (top), reaction condition: MAH (2.452 g), St (2.60 g), and AIBN (0.0329 g), reaction temperature 70 ± 1°C**.** TEM micrographs of the PMV copolymer microspheres prepared with stirring, reaction time: (B) 3 hours; (C) 4 hours. Preparation conditions: [MAn] = [VAc] = 1.0 M; BPO 0.8 wt% relative to monomers; temperature, 80 ± 1°C.

**
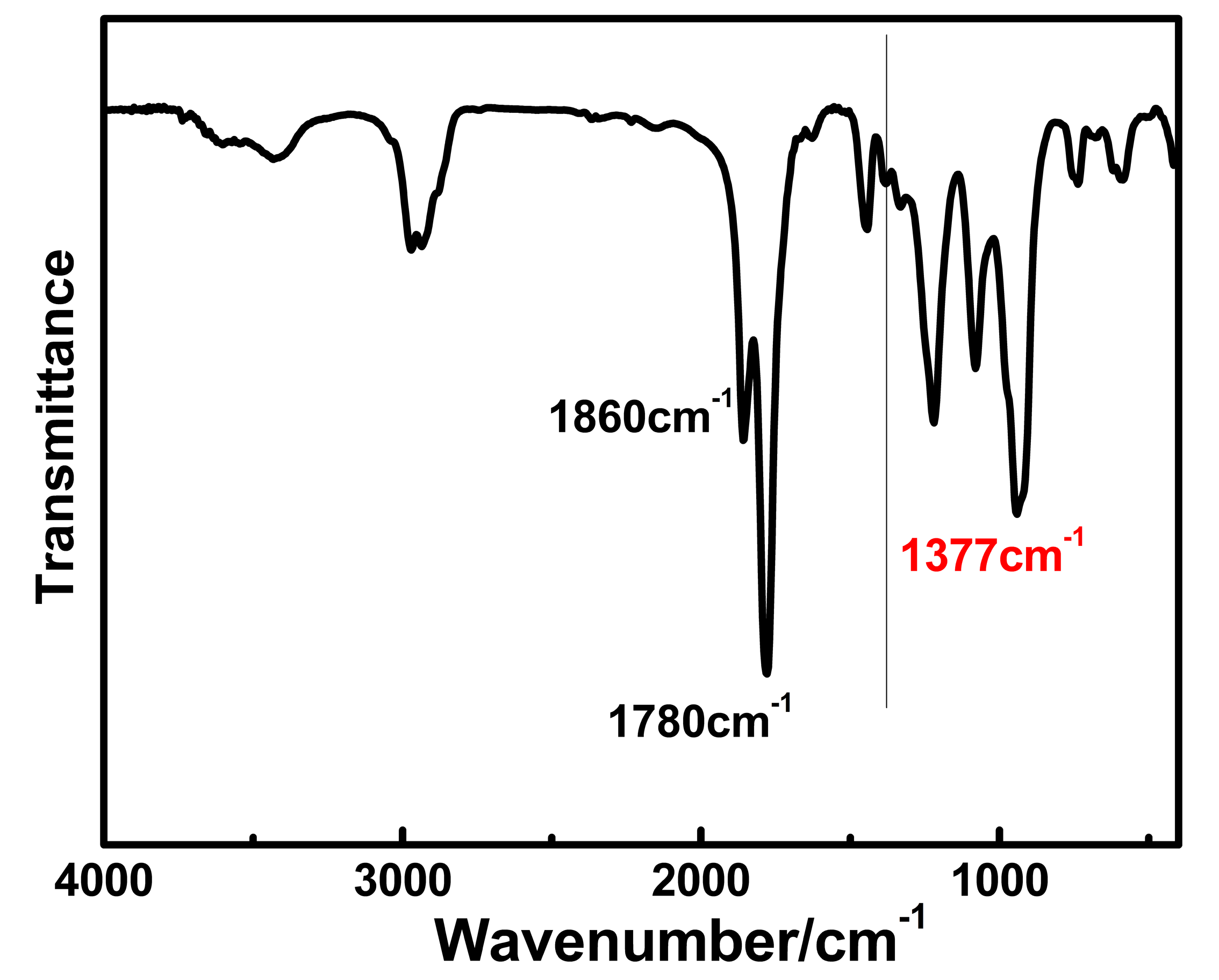
**

**Figure S4.** The FT-IR spectrum of the poly (maleic anhydride-alt-1-butene).

**Table S1.** The particle size, particle size distribution and composition of microspheres formed with different molar feed ratio of MAH/St and monomers concentrations.^#^

| Experiments | Molar feed ratio MAH/St | *D*_n_ (nm) | *D*_w_ (nm) | PDI | Copolymer composition  MAH mol% |
| --- | --- | --- | --- | --- | --- |
| A | 4:1 | 1076 | 1108 | 1.030 | 50.5 |
| B | 3:1 | 947 | 968 | 1.022 | 50.3 |
| C | 2:1 | 734 | 741 | 1.010 | 50.1 |
| D | 1:1 | 538 | 543 | 1.009 | 50.1 |
| E | 1:2 | 712 | 717 | 1.007 | 49.6 |
| F | 1:3 | 821 | 827 | 1.007 | 49.5 |
| G | 1:4 | 950 | 958 | 1.008 | 49.3 |

#: A~D, the concentration of St was fixed at 1.0 mol/.L, and the concentration of MAH varied from 4 mol/L to l mol/L; E~G, the concentration of MAH was fixed at 1 mol/L, and the concentration of St varied from 2 mol/L to 4 mol/L. The reaction media was IA, and the reaction temperature was 70 ± 1°C. The copolymer composition was quantitatively analyzed by titration.

**Table S2.** Some parameters of PMS particles obtained at different polymerization time.

| Reaction time (min) | M_n_^*^  (KDa) | M_w_^†^  (KDa) | M_z_^‡^  (KDa) | PDI ^§^  M_n_/M_w_ | Y_p_^‖^  (%) | D_n_  (nm) | PDI |
| --- | --- | --- | --- | --- | --- | --- | --- |
| 10 | 232 | 1250 | 3534 | 5.378 | 2.36 | 197 | 1.058 |
| 15 | 243 | 1306 | 2926 | 5.367 | 9.64 | 292 | 1.050 |
| 20 | 218 | 1135 | 2814 | 5.196 | 20.1 | 346 | 1.034 |
| 25 | 199 | 1122 | 2822 | 5.635 | 28.89 | 390 | 1.022 |
| 30 | 169 | 959 | 2706 | 5.660 | 38.19 | 428 | 1.014 |
| 35 | 157 | 943 | 2701 | 6.000 | 47.73 | 460 | 1.014 |
| 50 | 138 | 930 | 2723 | 6.738 | 64.51 | 507 | 1.010 |
| 60 | 130 | 947 | 2753 | 7.276 | 71.07 | 521 | 1.009 |
| 90 | 104 | 780 | 2635 | 7.518 | 80.10 | 538 | 1.006 |
| 120 | 96 | 702 | 2588 | 7.276 | 82.00 | 539 | 1.009 |

^*^ The number average molecular weight. ^†^ The weight average molecular weight. ^‡^ The Z average molecular weight. ^§^ The molecular weight distribution. ^‖^ Yield of polymer particles.

Reaction conditions: MAH (7.356 g), St (7.8 g), IA (135mL) and AIBN (0.0987 g), reaction temperature 70 ± 1°C. No agitation was used during polymerization. At different reaction time, 2mL reaction solution was drawn from the reaction system.

**Table S3.** Effect of solvents on alternative copolymerization of St and MAH

| **Experiment** | **Solvent** | **Solubility parameter δ^*^**  **MPa^1/2^** | **ε^†^** | **η^‡^**  **(mPa.s)** | **Result** |
| --- | --- | --- | --- | --- | --- |
| 1 | IA | 16.0 | 4.63 | 0.872 | dispersion |
| 2 | ethyl butyrate | 17.4 | 5.2 | 0.613 | dispersion |
| 3 | amyl acetate | 17.4 | 4.75 | 0.924 | dispersion |
| 4 | n-Butyl acetate | 17.5 | 5.01 | 0.734 | dispersion |
| 5 | xylene | ～18.0 | 2.266 | ～0.754 | particles-gel |
| 6 | toluene | 18.2 | 2.24 | 0.587 | particles-gel |
| 7 | ethyl acetate | 18.6 | 6.02 | 0.449 | gel |
| 8 | ethyl benzoate | 16.8 | 5.98 | 1.956 | dispersion |
| 9 | butanone | 19.0 | 18.51 | 0.423 | solution |
| 10 | acetone | 20.3 | 20.70 | 0.316 | solution |
| 11 | 1，4-dioxane | 20.5 | 2.209 | 1.3 | solution |
| 12 | ethyl alcohol | 26.0 | 25.7 | 0.595 | solution |

^*^ Solubility parameter (25°C). ^†^ Dielectric constant (20°C). ^‡^ Solvent viscosity (20°C)

Reaction conditions: MAH (2.452 g), St (2.60 g), and AIBN (0.0329 g), polymerization for 6 hours at temperature 70 ± 1°C. No agitation was used during polymerization.

**Notes:** It can be seen from the Table S3 that among three parameters, δ, ε and η, only solubility parameter δ demonstrates a regular relationship with the behavior of the precipitate polymerization.

**Copolymerization of typical olefinic compounds and C5/C9 fractions with MAH**

All of the polymerization procedures, separation and characterization are same as those described previously. The only difference is that, for the compounds with low C numbers such as butadiene, isoprene, cyclopentene, the reactor was **sealed** after purging N_2_ and the polymerization was performed under sealed state at the fixed temperature. Detailed information about the solvent, initiator (concentration), the ratio of olefinic compound to MAH and their concentration, polymerization temperature and time can be found in the corresponding notes.

**Table S4** The composition of C_5_ mixture

| Species | Component | Percentage content |
| --- | --- | --- |
| Dienes | Isoprene, cyclopentadiene, dicyclopentadiene  1,4-pentadiene, 1,3-pentadiene | 48.41% |
| Olefines | 1-pentene, 2-pentene, cyclopentene, 2-methyl-1-butene, 2-methyl-2-butene | 12.06% |
| Alkanes | Isopentane, n-pentane, cyclopentane, 2-methylpentane,  n-hexane, methylcyclopentane | 22.66% |
| Alkynes | 2-butyne, 3-penten-1-yne | 0.98% |
| Others | C4 mixture, benzene, dimers, unknown component | 15.89% |

**Table S5.** The composition of C9 fraction

| Component | Percentage content |
| --- | --- |
| **Cyclopentadiene** | 4.30 |
| **Methylcyclopentadiene** | 1.42 |
| between | 0.06 |
| **Toluene** | 0.28 |
| between | 0.13 |
| **Ethylbenzene** | 1.25 |
| **m-Xylene**, **p-Xylene** | 4.36 |
| **Styrene** | 11.35 |
| **o-Xylene** | 2.70 |
| between | 0.23 |
| **Isopropylbenzene** | 0.11 |
| between | 0.09 |
| **Allylbenzene** | 0.56 |
| **Propylbenzene** | 0.42 |
| between | 0.06 |
| **Methyl ethylbenzene** | 2.03 |
| between | 0.51 |
| **Trimethylbenzene** | 1.00 |
| between | / |
| **Trimethylbenzene** + **Vinyltoluene** | 3.33 |
| between | 1.31 |
| **Indan** | 34.23 |
| **Indene** | 1.49 |
| **Dihydrodicyclopentadiene** | 3.32 |
| **Methyldicyclopentadiene** | 6.20 |
| **Dihydro methyldicyclopentadiene** | 5.04 |
| **Tetrahydrodicyclopentadiene** | 4.19 |
| **Tetrahydro methyldicyclopentadiene** | 0.31 |
| between | 0.27 |
| **Dihydro dimethyldicyclopentadiene** | 1.77 |
| after | 7.71 |
